# Dataset for determining rational taxation value with incompatible criteria of economic efficiency and equity

**DOI:** 10.1016/j.dib.2019.104532

**Published:** 2019-09-17

**Authors:** Elvir Munirovich Akhmetshin, Nataliya Stepanovna Plaskova, Iuliana Ivanovna Iusupova, Natalia Alekseevna Prodanova, Alexey Nikolaevich Leontyev, Vladimir Lvovich Vasilev

**Affiliations:** a Kazan Federal University, Elabuga Institute of KFU, Elabuga, Russian Federation; b Plekhanov Russian University of Economics, Moscow, Russian Federation; c I.M. Sechenov First Moscow State Medical University, Moscow, Russian Federation; d Baltic International Academy, Riga, Latvia

**Keywords:** Data set, Analysis of taxation, Optimization of taxation, Criterion of economic efficiency, Justice criterion, Successive assignment method, Simultaneous increase of efficiency and equity of taxation, Kendall coefficient of concordance

## Abstract

This article is essentially a dataset necessary for analysing the taxation. The data analysis has allowed to determine the optimal taxation model, when the criteria of economic efficiency and equity are incompatible. The dataset has allowed the use of the method of successive concessions in tax optimization. The practical significance of the dataset lies in the ability to simultaneously improve the efficiency and equity in taxation.

The dataset was obtained by using the method of expert estimates. A group of experts was asked to rank the taxes established by the Tax Code of the Russian Federation, in descending order of importance. Only strict rankings were allowed. The consistency of expert opinion was evaluated using the Kendall coefficient of concordance.

The data set was supplemented with the expert ranking data of the basic principles of taxation, such as the principle of equity; the principle of certainty and accuracy of taxes; the principle of ease of tax collection for taxpayers; the principle of efficiency; the principle of commitment.

The dataset can be used in the future to determine a rational amount of taxation depending on the established criteria.

**Table:** Specifications Table

Subject	Economics, Econometrics and Finance
Specific subject area	Taxes, tax system, taxation, state budget revenues, national fiscal policy
Type of data	Table
How data were acquired	The dataset on the types of taxes was obtained by analysing open sources of information, official Internet sites of public administration and statistics. Part of the data was obtained through an expert survey on the importance and significance of taxes and taxation criteria.
Data format	Analysed
Parameters for data collection	The data were collected according to the parameter of assessing the importance and significance of 15 types of taxes stated in the budget legislation. Another parameter was presented by 5 principles of taxation. The most important parameter for data collection was the interrelation of the principles of equity of the tax system and its efficiency. The method of expert assessments was used.
Description of data collection	22 expert surveys were conducted and processed to collect and compile the data set. The experts had experience in the field of tax administration and were the developers of ways to improve the tax system. The age of the experts was 25–45. The gender composition was mixed. The surveys were conducted anonymously. When processing the results, in one of the cases a lack of consensus was revealed, which led to the division of the expert group into two subgroups. The experts were divided into two groups of 14 and 8 people respectively. To identify the consistency of expert opinions, the Kendal concordance index was calculated. The results of the expert survey and checking their level of consistency showed a high credibility of the data set. The survey was conducted at the end of 2018, however, all the experts were informed in advance about the forthcoming changes in the Tax legislation that would be put into effect and planned for introduction in 2019 (such as the tax on self-employed people and a number of resort and hotel fees). It should be noted that the experts had not had experience in the practical use of these changes or feedback on their application by the time of the survey.
Data source location	Data source: survey readings of the 22 experts. The survey was conducted among experts from several cities of the Russian Federation: Moscow, Kazan (Republic of Tatarstan).
Data accessibility	With the article
Related research article	A. Leontyev, Analytical research of the tax optimization impact on the budget revenues, Accounting in construction organizations, 3 (181), 2019, 76–86 [1].

**Table:** 

**Value of the Data** • The data set presented in this article will be useful for analyzing and improving the national tax policy at all levels of power. The dataset will optimize the taxation • The dataset can be used in the development of expert decision-making systems in the field of taxation and fiscal policy at the level of municipalities, regions and the federal center. The dataset will also be useful for developing expert decision-making systems for private business and entrepreneurs • The dataset will make it possible to compile ratings on the importance and significance of the taxation criteria within local or regional budgets. In turn, the ratings will allow to determine the optimal policy of taxation depending on the specific conditions of the local or regional socio-economic environment.

## 1 Data

To obtain the data set, 22 experts in the field of the state taxes, tax policy and tax system were interviewed. The task of the experts was to determine the significance of the tax for the socio-economic system as a whole. It was proposed to consider a total of 15 taxes [2]:

1. Mineral Extraction Tax (Tax Code of the Russian Federation, part II, chapter 26)
2. Value Added Tax (VAT) (Tax Code of the Russian Federation, part II, chapter 21)
3. Corporate Profit Tax (Tax Code of the Russian Federation, part II, chapter 25)
4. Excise taxes (Tax Code of the Russian Federation, part II, chapter 22)
5. Corporate Property Tax (Tax Code of the Russian Federation, part II, chapter 30)
6. Individual Property Tax (Tax Code of the Russian Federation, part II, chapter 23)
7. Gambling Tax (Tax Code of the Russian Federation, part II, chapter 29)
8. Transport Tax (Tax Code of the Russian Federation, part II, chapter 28)
9. Land Tax (Tax Code of the Russian Federation, part II, chapter 31)
10. Regular payments for mineral resource use (Clause 43 of the Law of the Russian Federation “On Subsoil")
11. Charges for the use of aquatic biological resources and water tax (Tax Code of the Russian Federation, part II, chapter 25.1 and 25.2)
12. Trading fee (Tax Code of the Russian Federation, part II, chapter 33)
13. Individual Property Tax (Tax Code of the Russian Federation, part II, chapter 32)
14. Levies for the use of fauna (Tax Code of the Russian Federation, part II, chapter 25.1)
15. Real property taxation at cadastral value (Tax Code of the Russian Federation, part II, Clause 403)

The initial data set is presented in Table 1. The data were obtained by interviewing the experts. A total of 22 experts were interviewed. The experts were to arrange the proposed taxes in order of decreasing their significance for the socio-economic system from 1 to 15: 1 — the most important tax, 15 — the least important tax. The survey data and the data set obtained are shown in Table 1.

**Table 1:** Primary (raw) data collected from the expert survey for tax ranking.

Expert number	Ranking by the importance of the Russian Federation taxes														
	Value Added Tax (VAT)	Mineral Extraction Tax	Land tax	Regular payments for mineral resource use	Trading fee	Charges for the use of aquatic biological resources and water tax	Excise taxes	Tax on personal income	Real property taxation at cadastral value	Levies for the use of fauna	Individual Property Tax	Corporate Property Tax	Gambling Tax	Corporate Profit Tax	Transport Tax
1	2	1	11	12	10	9	3	4	13	14	15	5	6	7	8
2	2	1	11	12	8	10	4	9	15	13	14	3	7	5	6
3	3	7	8	6	1	14	9	2	15	10	13	11	12	5	4
4	10	3	11	4	12	13	5	9	6	15	7	8	1	2	14
5	3	1	10	9	7	13	8	12	15	11	14	5	4	2	6
6	2	1	9	12	11	10	3	5	13	14	15	4	6	8	7
7	2	1	11	12	13	9	4	7	10	14	15	3	5	6	8
8	1	3	12	10	13	9	2	5	15	14	11	4	6	7	8
9	3	1	9	13	14	10	4	6	15	12	11	5	7	2	8
10	2	1	10	11	13	12	3	4	14	15	9	5	6	8	7
11	2	1	11	13	12	10	5	6	14	8	15	4	7	3	9
12	4	1	11	14	12	13	3	9	10	15	8	5	6	2	7
13	3	2	10	11	14	12	4	7	9	13	15	6	5	1	8
14	2	1	8	10	12	11	4	9	15	14	13	6	5	3	7
15	2	1	7	8	10	9	3	6	14	12	15	5	11	4	13
16	2	1	12	11	10	13	5	6	15	14	9	3	8	4	7
17	2	3	11	10	12	9	4	8	14	13	15	5	6	1	7
18	4	2	12	13	11	9	3	8	10	15	14	7	5	1	6
19	2	4	13	14	10	8	3	1	15	12	11	9	6	5	7
20	1	3	9	10	12	11	4	2	13	15	14	5	8	6	7
21	2	3	10	8	13	12	5	1	14	11	15	7	6	4	9
22	2	1	11	10	12	9	6	5	15	14	13	4	7	3	8

To form the dataset on the importance and significance of taxation, the same group of experts was asked to rank the following basic principles of taxation:

- the principle of equity;
- the principle of certainty and accuracy of taxes;
- the principle of ease of tax collection for taxpayers;
- the principle of efficiency;
- the principle of commitment.

As in the previous survey, it was necessary to rank the principles of taxation by degree of importance, assigning them numbers from 1 to 5, where 1 is the most significant principle, 5 is the least significant principle. Only strict rankings were allowed. The survey data and the final data set are shown in Table 2.

**Table 2:** Primary (raw) data collected from a survey of the group of experts to rank taxation principles.

Expert number	Ranking on the importance of taxation principles				
	Principle of equity	Principle of certainty and accuracy of taxes	Principle of ease of tax collection for taxpayers	Principle of efficiency	Principle of commitment
1	4	1	5	3	2
2	1	4	5	3	2
3	3	2	5	4	1
4	1	2	5	3	4
5	4	1	5	3	2
6	1	2	4	3	5
7	2	1	5	3	4
8	1	2	5	4	3
9	3	1	4	5	2
10	1	3	5	4	2
11	4	3	5	2	1
12	1	2	5	3	4
13	2	1	4	3	5
14	3	2	5	4	1
15	1	2	4	4	3
16	2	1	3	5	4
17	1	2	5	4	3
18	2	3	4	5	1
19	1	2	4	5	3
20	2	4	5	3	1
21	1	3	4	5	2
22	1	2	5	4	3

Since humanity has defined the concept of equity for itself, it has been unsuccessfully trying to give an accurate description of this category. Moreover, equity is one of the basic principles of taxation. Since the time of Adam Smith to the present day, economic science has not yet definitively decided on the essence and content of this term. The term efficiency of tax policy appeared much later and immediately took an antagonistic position in relation to equity. Very often there is an opinion that these concepts are mutually exclusive and it is almost impossible to resolve this antagonism, i.e. one will always have to choose between equity and efficiency. However, efficiency in a tax system is usually understood as more beneficial for the state, whereas equity - for taxpayers.

The incompatibility of the requirements of equity and efficiency can be described as follows: it is known that raising taxes on the one hand increases the revenue side of the budget, and on the other hand, can lead to bankruptcy of enterprises due to excessive tax burden. In this case, the state on the one hand wins, receiving a large amount from a particular taxable entity, on the other - loses due to a decrease in the number of taxable entities, loss of jobs and increase in number of unemployed.

## 2 Experimental design, materials, and methods

The database was formed in three stages.

**At the first stage**, methods of statistics and expert evaluation were applied. To date, a large number of different expert-survey-based decision-making methods have been developed.

The main stages of solving a problem of determining weight coefficients by expert assessment methods are presented as follows:

1. Setting the research task - determining priority (ranking) of relative single indicators characterizing the principles of tax policy, namely, commitment, equity, efficiency, certainty and accuracy, ease of tax collection for taxpayers, as well as ranking taxes provided by the tax code of the Russian Federation.
2. Choosing the method of obtaining expert information and methods of its processing. Direct questioning of experts was chosen as the method of obtaining expert information, and the decision making on vector criteria as the method of processing. At the same time, various possible decisions on preference are ranked directly (the indicators are placed according to importance) for use in the above-considered method of successive concessions.
3. The expert group formation. The group of experts was formed from among professors, lecturers and employees of economic departments of universities of Russia, as well as from among employees engaged in accounting services for companies. The total number of experts was 22.
4. Collecting the expert information. The experts were given questionnaires. A strict ranking of elements was allowed, in which different ranks were assigned to different elements. In order to avoid conformism, the interaction of experts with each other during the survey was excluded.
5. Processing and analysis of the information received (See Table 3, Table 4, Table 6).

**Table 3:** Processing of the data set for tax rating.

Expert number	Ranking by the importance of the Russian Federation taxes														
	Value Added Tax (VAT)	Mineral Extraction Tax	Land tax	Regular payments for mineral resource use	Trading fee	Charges for the use of aquatic biological resources and water tax	Excise taxes	Tax on personal income	Real property taxation at cadastral value	Levies for the use of fauna	Individual Property Tax	Corporate Property Tax	Gambling Tax	Corporate Profit Tax	Transport Tax
$x^{j}$	58	43	227	233	242	235	94	131	289	288	281	119	140	89	171
$x^{j}-\bar{x}$	−118	−133	51	57	66	59	−82	−45	113	112	105	−57	−36	−87	−5
${\left(x^{j}-\bar{x}\right)}^{2}$	13924	17689	2601	3249	4356	3481	6724	2025	12769	12544	11025	3249	1296	7569	25

**Table 4:** Processing of the data set for taxation principles ranking.

Expert number	Ranking on the importance of taxation principles				
	Principle of equity	Principle of certainty and accuracy of taxes	Principle of ease of tax collection for taxpayers	Principle of efficiency	Principle of commitment
${\text{x}}^{\text{j}}$	42	46	101	82	58
${\text{x}}^{\text{j}}-\bar{\text{x}}$	−24	−20	35	16	−8
${\left({\text{x}}^{\text{j}}-\bar{\text{x}}\right)}^{2}$	576	400	1225	256	64

**Table 5:** Primary (raw) data collected from the results of a survey of the first subgroup of experts for taxation principles ranking.

Expert number	Ranking on the importance of taxation principles				
	Principle of equity	Principle of certainty and accuracy of taxes	Principle of ease of tax collection for taxpayers	Principle of efficiency	Principle of commitment
1	1	2	5	3	4
2	1	2	4	3	5
3	2	1	5	3	4
4	1	2	5	4	3
5	1	2	5	3	4
6	2	1	4	3	5
7	1	2	4	4	3
8	2	1	3	5	4
9	1	2	5	4	3
10	1	2	5	4	3
11	3	1	4	5	2
12	2	3	4	5	1
13	1	2	4	5	3
14	1	3	4	5	2

**Table 6:** Processing of the data set for the taxation principles ranking in the first subgroup of experts.

Expert number	Ranking on the importance of taxation principles				
	Principle of equity	Principle of certainty and accuracy of taxes	Principle of ease of tax collection for taxpayers	Principle of efficiency	Principle of commitment
${\text{x}}^{\text{j}}$	20	26	61	56	46
${\text{x}}^{\text{j}}-\bar{\text{x}}$	−22	−16	19	14	4
${\left({\text{x}}^{\text{j}}-\bar{\text{x}}\right)}^{2}$	484	256	361	196	16

**At the second stage** the quality of the dataset obtained was checked. For a precise definition of the necessary combination of equity and efficiency, given the unavoidable contradictions of these criteria, it was suggested that they be ranked. There are various methods for ranking variables to determine significance and subsequent operations on these variables. Among them is The Kendall Rank Correlation Coefficient [3]. As Jeremy M. G. Taylor describes: Kendall's tau is commonly used nonparametric methods of detecting associations between two variables. Their use is usually restricted to a single block [4]. The Kendall rank correlation coefficient evaluates the degree of similarity between two sets of ranks given to a same set of objects. This coefficient depends upon the number of inversions of pairs of objects which would be needed to transform one rank order into the other [5]. The use of this coefficient seems possible to be included in processing the primary information obtained in the process of data collection, both for statistical data and for those obtained by the method of expert estimates [6], [7], [8], [9].

The group of 22 experts was asked to determine the significance and importance of taxes according to different taxation principles. It was necessary to rank the elements a_1_, a_2_, a_3_, … a_15_ and b_1_, b_2_, b_3_, … b_5_. Various ranking options were obtained in the process. Ranks of the elements were introduced for the data processing. x_j_ rank of a_j_ element indicates the number of this element in the ranking list. In this case, the element in the first place has a rank equal to one. For example, if the elements in the ranking list are distributed in the following sequence: a_3_, a_1_, a_2_, then we have *х*_*3*_ = *1*, *х*_*1*_ = *2*, *х*_*2*_ = *3*.

The dataset characterizing the priority of the existing taxes, is presented in Table 1. The data are compiled as a sequence of ranks:

(1) $$\begin{array}{c} x_{11},x_{12},\ldots x_{1n}\\ x_{21},x_{22},\ldots x_{2n}\\ \ldots \;\ldots \;\ldots \;\ldots \\ x_{m1},x_{m2},\ldots x_{mn} \end{array}\}$$

The processed data characterizing the priority of existing taxes are presented in Table 3.

Processing of data characterizing the priority of the taxation principles is presented in Table 4.

This ranking in many criteria represent a fairly objective picture, especially for those taxes, the sum of the ranks x^j^ of which differs significantly from the closest ones. In this case, the taxes with the sum of the ranks x^j^ differing slightly from each other, probably can exchange places, with a more careful selection of experts. For example, regular payments for mineral resource use have a sum of 233, while fees for the use of aquatic biological resources and water tax are 235. A similar picture is observed when analyzing the amount of ranks of fees for using fauna and real estate cadastral value - here the difference is only one (288 and 289, respectively).

The assessment of the consistency of expert opinions was made using rank correlation coefficients, for which a special concordance (consistency) coefficient was used. We will use the concordance coefficient W proposed by Kendall.

To do this, we determine the sum of the ranks by the following expression:

(2) $$x^{j}=\overset{m}{\underset{i=1}{\sum }}x_{ij,j=\bar{1,n}}$$

where:

*m* – is the number of experts;

*n* – is the number of ranking elements.

The values of $x^{j}$ for the survey related to the ranking of taxes are given in Table 3. Since the survey allowed only strict ranking, the average value of the sum of ranks $\bar{x}$ is determined by the formula:

(3) $$\bar{x}=\frac{1}{n}\overset{m}{\underset{i=1}{\sum }}\overset{n}{\underset{j=1}{\sum }}x_{ij}=\frac{m\left(n+1\right)}{2}$$

For the data given in Table 3, $\bar{x}$ = 176. Determine the sum of squares of deviations of $x^{j}$ from the average $\bar{x}$ by the formula:

(4) $$S_{w}=\overset{n}{\underset{j=1}{\sum }}{\left(x^{j}-\bar{x}\right)}^{2}$$

For the data given in Table 3, $S_{w}=102526$.

For strict ranking, the sum of the squares of their deviations from the average $\bar{x}=0,5m\left(n+1\right)$ will make:

(5) $$S=\overset{n}{\underset{j=1}{\sum }}{\left(jm-\frac{m\left(n+1\right)}{2}\right)}^{2}=\frac{1}{12}m^{2}n\left(n^{2}-1\right)$$

For the case in question, S=135520.

The concordance coefficient is determined by the formula:

(6) $$W=\frac{Sw}{S}$$

For Table 1, W=0,76.

The dataset obtained in the course of the expert survey should be considered consistent if the value of the concordance coefficient is greater than 0.6. Therefore, the expert opinion on this issue should be considered as agreed.

The analysis of the dataset from Table 4 was carried out as follows. The mean value of the sum of the ranks $\bar{x}$, determined by formula (3), is $\bar{x}$ = 66. The sum of the squares of deviations x^j^ (calculated by formula (2)) from the average value of $\bar{x}$ (calculated by formula (3)) was determined by formula (4) and amounted to S_w_ = 2521. The sum of the squares of their deviations from the mean value of $\bar{x}$ was determined by formula (5). For the case under consideration, S = 4840. The coefficient of concordance (6) for Table 4 was W = 0.52.

As already mentioned, the expert opinion is agreed if the value of the concordance coefficient is greater than 0.6. Since in this case the value of V < 0.6, then for further data collection it is advisable to divide the experts, according to their opinions (on the degree of agreement) into two groups (the number of groups may be larger).

**At the third stage,** the data set was analyzed for the tax policy principles priority. The problem is that the principles of tax policy may be limitedly compatible. The most controversial pair of principles is “equity - efficiency” of the tax policy.

In a number of previously published papers [10], [11], an optional optimization method (tax prism method) was proposed for determining the optimal value of the tax burden. Also, various situations were considered there that may make it difficult to find the specified parameter. It was noted that the most difficult situation is the incompatibility of the requirements described by any relative single indicators formed during the construction of the combined diagram.

The use of the optimization method of successive concessions in the case of a non-removable incompatibility of the requirements of the “equity - efficiency” system is inevitably associated with the ranking of the relative single indicators, as well as the taxes associated with them, in descending order of their significance [12]. If accurate statistical data is available in individual cases, a probabilistic approach can be used for ranking, or the expert estimation method.

To resolve the contradiction of justice and efficiency, various types of research were conducted, during which it was noted that when the state has no other opportunity to improve the situation of single taxpayers without simultaneously worsening the situation of others, one may consider achieving Pareto optimality or achieving Pareto efficiency [13].

The concept of “utility” is used in the literature on optimal taxation, no less, or maybe even more often than the concept of “income”. K. Heidi has specifically investigated this phenomenon [14].

There is a widespread concept of proportional income taxation as of meeting the criteria for optimal taxation. J. Mirrlis considered it an axiom that earned incomes of industrial and labor origin in the view of ensuring social welfare should not be taxed on a progressive scale [15]. However, the optimal taxation of total income in any system of social criteria according to J. Bentham and J. J. Rawls should be progressive [16]. In J. Mirrlis's opinion the maximum proportional tax rate is to reach 60%, while in the opinion of his compatriot J. Merli it should be no more than 20%. Obviously, from the standpoint of the priority of efficiency, the state should choose a proportional, and even better, a lump-sum tax, but there can be no question of any equity in this case [17].

For the dataset presented in Table 3, the data were divided into two groups to identify the importance and significance of the principles of taxation. The first group included 14 experts (Table 5), the second 8 (Table 8).

For the group of 14 experts (according to Table 5 and Table 6): $\bar{x}$ = 42, $S_{w}=1313$, S=1960. The concordance coefficient (6): W=0,67 (i.e.more than 0.6). Therefore, the expert opinion is agreed. In accordance with it, the principles of taxation are distributed in terms of importance in the order indicated in Table 7.

**Table 7:** The distribution of the taxation principles in terms of the importance after processing the opinions of the first subgroup of experts.

	Taxation principle
1.	equity
2.	certainty and accuracy of taxes
3.	commitment
4.	efficiency
5.	ease of tax collection for taxpayers

For the group of 8 experts (according to Table 8 and Table 9): $\bar{x}$ = 24, S_w_ = 424, S = 640. The coefficient of concordance (6): W = 0.66 (i.e., more than 0.6). Therefore, the expert opinion is agreed. In accordance with it, the principles of taxation are distributed in terms of importance in the order indicated in Table 10.

**Table 8:** Primary (raw) data collected from a survey of the second subgroup of experts for taxation principles ranking.

Expert number	Ranking on the importance of taxation principles				
	Principle of equity	Principle of certainty and accuracy of taxes	Principle of ease of tax collection for taxpayers	Principle of efficiency	Principle of commitment
1	4	1	5	3	2
2	1	4	5	3	2
3	3	2	5	4	1
4	4	1	5	3	2
5	1	3	5	4	2
6	4	3	5	2	1
7	3	2	5	4	1
8	2	4	5	3	1

**Table 9:** Processing of the data set for the taxation principles ranking in the second group of experts.

Expert number	Ranking on the importance of taxation principles				
	Principle of equity	Principle of certainty and accuracy of taxes	Principle of ease of tax collection for taxpayers	Principle of efficiency	Principle of commitment
$x^{j}$	22	20	40	26	12
$x^{j}-\bar{x}$	−2	−4	16	2	−12
${\left(x^{j}-\bar{x}\right)}^{2}$	4	16	256	4	144

**Table 10:** The distribution of the taxation principles in terms of the importance after processing the opinions of the second subgroup of experts.

	Taxation principle
1.	commitment
2.	certainty and accuracy of taxes
3.	equity
4.	efficiency
5.	ease of tax collection for taxpayers

It should be noted that, despite the difference in opinions of the two subgroups of experts we had to make by splitting the whole group of experts, both subgroups have set the priority of the principle of equity of tax collection over the principle of efficiency.

It is worth noting that in the subgroup of 14 people experts with an academic background prevailed, while in the subgroup of 8 experts there were people mainly with practical and managerial experience.

The presented dataset suggests that, in order to improve tax policy, it is necessary not to hinder the efficient allocation of resources and to be fair to the various participants in the tax process.

Most often in reality, there are situations when the tax system improves the welfare of some people, worsening the situation of others. The criterion of optimal taxation - the criterion of tax equity is to help to decide whether the given tax system is acceptable or unacceptable for the society.

A fair tax system, as a rule, is focused on the enforced use of the most significant tax bases with minimal price elasticities of the demands and proposals. In this case, the loss of efficiency with an increase in the corresponding taxes will be the lowest.

## References

[1] Leontyev A.N. (2019). Analytical research of the tax optimization impact on the budget revenues. Account. Const. Comp..

[2] Tax Code of the Russian Federation Part II dated 05.08.2000 #117-FZ (2000, August 05). Legal reference system "consultant plus". http://www.consultant.ru/document/cons_doc_LAW_28165/.

[3] Kendall M.G. (1948). Rank Correlation Methods.

[4] Taylor J. (1987). Kendall's and spearman's correlation coefficients in the presence of a blocking variable. Biometrics.

[5] Abdi H., Williams L.J., Valentin D. (2013). Multiple factor analysis: principal component analysis for multitable and multiblock data sets. Wiley Interdiscip. Rev. Comput. Stat..

[6] Puth M., Neuhäuser M., &Ruxton G.D. (2015). Effective use of spearman's and kendall's correlation coefficients for association between two measured traits. Anim. Behav..

[7] Chicco D., Ciceri E., Masseroli M. (2015). Extended Spearman and Kendall Coefficients for Gene Annotation List Correlation.

[8] Szmidt E., Kacprzyk J. (2011). The spearman and Kendall rank correlation coefficients between intuitionistic fuzzy sets.

[9] Croux C., Dehon C. (2010). Influence functions of the spearman and Kendall correlation measures. Stat. Methods Appl..

[10] Leontyev A., Verovska L. (2017). Use of the tax prism method when forming tax part of the budget. Econ. Cult..

[11] Leontyev A., Verovska L. (2018). Creation of the optimal tax part of the budget by using the tax prism method. International Scientific Symposium “Economics, Business & Finance”, Proceedings 2018.

[12] Gaube T. (2007). Optimum taxation of each year's income. J. Public Econ. Theory.

[13] Stiglitz J.E. (1987). Pareto Efficient and Optimal Taxation and the New New Welfare Economics.

[14] Heidi К., Devere M.L. (2001). Optimal Taxation as a Guideline of Tax Policy. Economics of Tax Policy. Transl. From English.

[15] Mirrlees J.A. (1971). An exploration in the theory of optimum income taxation. Rev. Econ. Stud..

[16] Andryushchenko V. (2000). Financial Idea of the West in the 20th Century: (Theoretical Conceptual and Scientific Problems of State Finance). Lviv: Kamenyar, 2000.

[17] Mirrlees J.A. (2011). An exploration in the theory of optimum income taxation. Welf. Incent. Tax..

